# Magnetoelectric Spin Wave Modulator Based On Synthetic Multiferroic Structure

**DOI:** 10.1038/s41598-018-28878-w

**Published:** 2018-07-18

**Authors:** Michael Balinskiy, Andres C. Chavez, Anthony Barra, Howard Chiang, Gregory P. Carman, Alexander Khitun

**Affiliations:** 1 Department of Electrical and Computer Engineering, University of California -Riverside, Riverside, California, 92521 USA; 2 Mechanical and Aerospace Engineering Department, University of California Los Angeles, Los Angeles, California, 90095 USA

## Abstract

We describe a spin wave modulator – spintronic device aimed to control spin wave propagation by an electric field. The modulator consists of a ferromagnetic film serving as a spin wave bus combined with a synthetic multiferroic comprising piezoelectric and magnetostrictive materials. Its operation is based on the stress-mediated coupling between the piezoelectric and magnetostrictive materials. By applying an electric field to the piezoelectric layer, the stress is produced. In turn, the stress changes the direction of the easy axis in the magnetostrictive layer and affects spin wave transport. We present experimental data on a prototype consisting of a piezoelectric [Pb(Mg_1/3_Nb_2/3_)O_3_]_(1-x)_ –[PbTiO_3_]_x_ substrate, and 30 nm layer of magnetostrictive Ni film, where the film is attached to a 30 nm thick Ni_81_Fe_19_ spin wave bus. We report spin wave signal modulation in Ni_81_Fe_19_ layer by an electric field applied across the piezoelectric layer. The switching between the spin wave conducting and non-conducting states is achieved by applying ±0.3 MV/m electric field. We report over 300% modulation depth detected 80 μm away from the excitation port at room temperature. The demonstration of the spin wave modulator provides a new direction for spin-based device development by utilizing an electric field for spin current control.

## Introduction

Spintronics is a rapidly evolving field of science aimed to benefit from the additional degree of freedom provided by spin^1,2^. It is an emerging approach towards novel computing devices^3^. Using spin in addition to charge makes it possible to build devices with sophisticated output characteristics, which can be utilized in Boolean as well as non-Boolean logic circuits^4^. One of the well-known examples is the modulator proposed by Datta and Das^5^. The operation of this device is based on the Rashba spin-orbit coupling effect, where the spin-polarized source-drain current is controlled by an electric field. The maxima and minima in transmission correspond to the parallel and antiparallel mutual orientation of spins in the channel and the drain. Thus, there are oscillations of the output electric current as a function of the gate voltage. The modulation is similar to the intensity modulation in the electro-optic modulators^5^. This work has encouraged a lot of research in the field of Spintronics leading to a variety of spin-based transistors^6,7^. The first working device made of InAs heterostructure was presented in 2009^8^. However, short spin diffusion length is one of the critical deep-seated problems in all spin-FETs. For instance, spin diffusion length in graphene does not exceed several micrometers at room temperature^9^. A possible solution may be found using collective spin phenomena (e.g., spin waves). Spin wave is a coherent precession of a large number of spins in magnetically ordered structures. A quantum of the spin wave is referred to as a magnon. Spin waves may preserve coherence over much longer distances compared to single electrons. For instance, spin wave coherence length in conducting ferromagnetic materials such as Ni_81_Fe_19_ may exceed tens of micrometers^10^. Spin wave coherence length in non-conducting ferrites (e.g., Y_3_Fe_2_(FeO_4_)_3_) exceed millimeters at room temperature^11^. The utilization of spin waves makes it possible to overcome constraints associated with the short spin diffusion length.

During the past decade, there has been a growing interest in spin wave logic devices and several working prototypes have been demonstrated^12–16^. There are different approaches to spin-wave signal modulation. For instance, the amplitude of the transmitted signal can be modulated by an external magnetic field^13^, via magnon-magnon scattering^14^, or via spin wave interference^17^. In all the above-mentioned works, the modulating signals are produced by electric-current carrying wires (e.g., micro-antennas). The use of electric current for spin wave modulation is not energetically efficient. For example, it takes about a 0.5 A electric current to modulate a spin wave signal in a Mach-Zehnder type interferometer reported in ref.^13^. It would be of great practical benefit to find a way to control spin wave transport by applying an electric field. For example, it is possible to excite spin waves by using voltage-controlled magnetic anisotropy^18–22^. The voltage-controlled magnetic anisotropy (VCMA) effect manifests itself as a variation of anisotropy of a thin layer of a conductive ferromagnet on a dielectric substrate under the influence of an external electric voltage. The application of a microwave voltage to a nano-sized VCMA gate in an ultrathin ferromagnetic nanowire results in the parametric excitation of a propagating spin wave. The required electric field is relatively high ~0.5 GV/m^19,20^. In this work, we consider synthetic multiferroics for voltage-controlled spin wave modulation, which may use much lower electric field for operation.

Multiferroics are a special type of material that possesses simultaneously electric and magnetic orders^23,24^. This translates to the possibility to generate a magnetic field by applying an electric field. There are only a few room temperature single phase multiferroic materials known today^24^, e.g. BiFeO_3_ and its derivatives. An alternative method for obtaining a structure with the magnetoelectric effect is a nanostructure consisting of two materials, a piezomagnetic film and a magnetostrictive film^25^. Magnetoelectric coupling arises as a combined effect of two: piezoelectricity and magnetostriction. An electric field applied to the piezoelectric produces stress, which, in turn, affects the magnetic properties of the magnetostrictive film. The combination of piezoelectric and magnetostrictive coupled materials may be considered as a *synthetic multiferroic or strain mediated multiferroic*. A two-layer magnetoelectric CoPd/PZT cell was experimentally demonstrated^26^, and the easy axis rotation of up to 150 degrees was observed. It was also experimentally demonstrated that the frequency of the microwave planar resonator consisting of yttrium iron garnet (YIG) and ferroelectric barium strontium titanate (BST) thin films can be both electrically and magnetically tuned^27^.

The original idea of combining a synthetic multiferroic with a spin wave bus was presented in ref.^28^. It was described a spin wave amplifier aimed to enhance the amplitude of the propagating spin wave via the magnetoelectric effect. According to numerical estimates, spin wave amplitude can be increased by several orders of magnitude. Later on, Cherepov *et al*.^29^ have demonstrated spin wave excitation and detection by synthetic multiferroics comprising PMN-PT/Ni/Py. Here, we describe a spin wave modulator based on a synthetic multiferroic. The rest of the paper is organized as follows. We describe the principle of operation of the modulator in Section II. We present experimental data obtained for PMN-PT/Ni and PMN-PT/Ni/Py structures in Section III. The obtained results are discussed in Sections IV.

## Modulator: Principle of Operation

**Figure 1:**
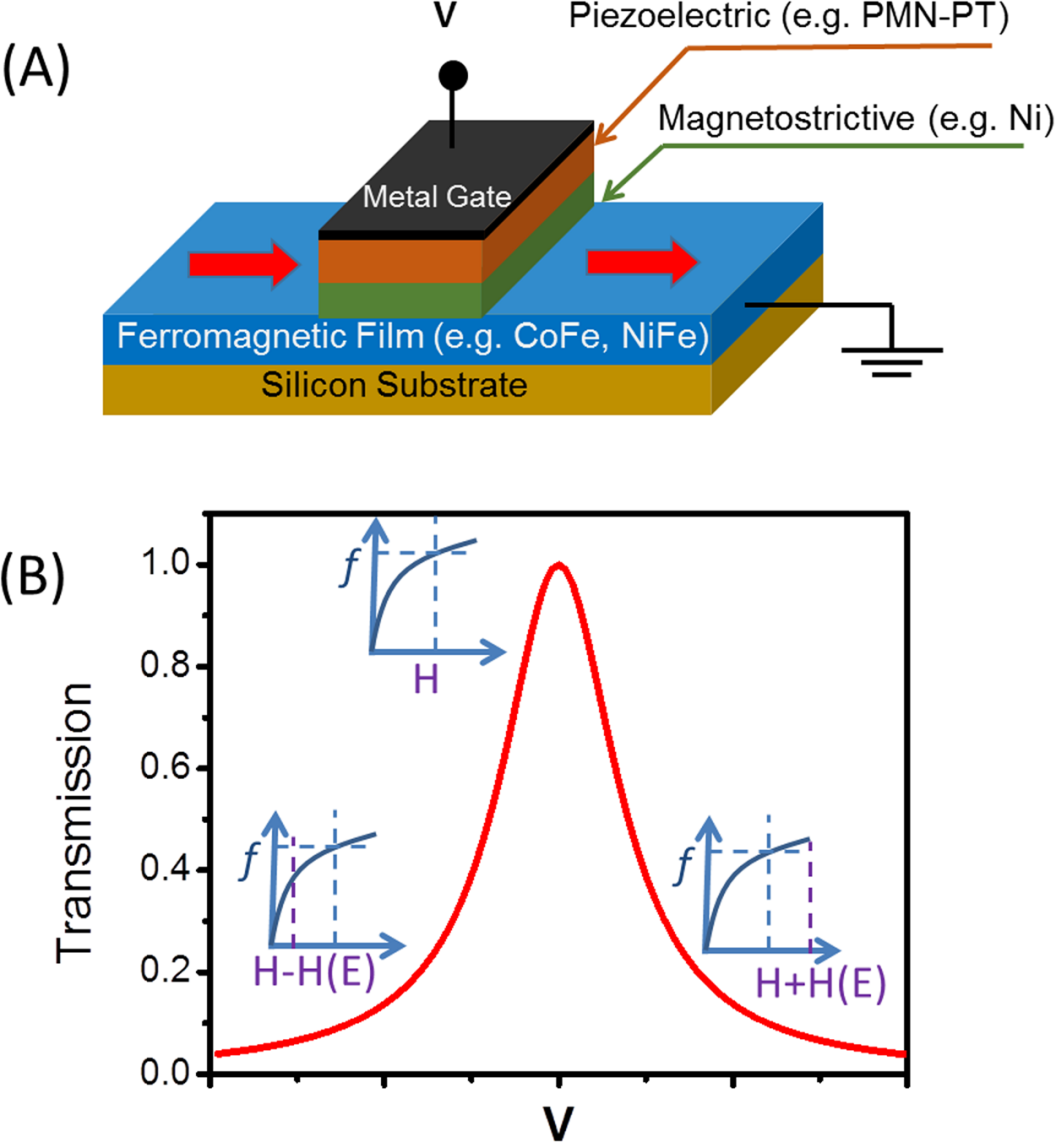
(**A**) Schematics of the spin-wave modulator. It consists of a semiconductor substrate (e.g. silicon), a conducting ferromagnetic film (e.g. CoFe, NiFe), a layer of magnetostrictive material (e.g. Ni), a piezoelectric layer (e.g. PMN-PT), and a metallic contact. The ferromagnetic film serves as a waveguide for spin waves –spin wave bus. (**B**) Output characteristics: spin-wave transmission as a function of the applied electric field. The insets illustrate the condition of switching between the spin wave conducting and non-conducting regimes.

The schematics of the spin wave modulator are shown in Fig. 1(A). From the bottom to the top, it consists of a semiconductor substrate (e.g., silicon), a conducting ferromagnetic film (e.g., CoFe, NiFe), a layer of magnetostrictive material (e.g., Ni), and a piezoelectric layer (e.g., 011 PMN-PT). The ferromagnetic film serves as a waveguide for spin waves – spin wave bus. We assume the thickness of the ferromagnetic film to be in the nanometer range to ensure in-plane magnetization (e.g., as shown in Fig. 1(A) red arrow). The metallic contact on the top of the piezoelectric layer and the conducting ferromagnetic film (ground plane) serve as a set of two electrodes to apply a voltage across the piezoelectric layer. In the absence of voltage (i.e. electric field), we assume that the piezoelectric layer does not influence spin wave propagation in the ferromagnetic layer. With an applied electric field *E*, the piezoelectric layer produces stress, which changes the easy-axis orientation in the magnetostrictive material. The spins of the magnetostrictive and ferromagnetic material are coupled via the exchange and dipole-dipole interactions. The magnetization change in the magnetostrictive layer influences the magnetization of the ferromagnetic layer. Thus, the stress produced by the piezo-element is equivalent to an additional external magnetic field $\overrightarrow{H}(E)$ in the ferromagnetic layer.

Spin wave propagation is well-described by the Landau-Lifshitz-Gilbert (LLG) equation^30,31^:

1 $$\frac{d\overrightarrow{m}}{dt}=-\,\frac{\gamma }{1+{\alpha }^{2}}\overrightarrow{m}\times [{\overrightarrow{H}}_{eff}+\alpha \overrightarrow{m}\times {\overrightarrow{H}}_{eff}],$$

where $\vec{m}=\vec{M}/{M}_{s}$ is the unit magnetization vector, *M*_*S*_ is the saturation magnetization, *γ* is the gyro-magnetic ratio, and *α* is the phenomenological Gilbert damping coefficient, ${\overrightarrow{H}}_{eff}$ is the effective magnetic field. The first term of equation (1) describes the precession of magnetization about the effective field and the second term describes its relaxation towards the direction of the field. The effective field ${\overrightarrow{H}}_{eff}$ can be expressed as follows^10^:

2 $${\overrightarrow{H}}_{eff}={\overrightarrow{H}}_{d}+{\overrightarrow{H}}_{ex}+{\overrightarrow{H}}_{a}+{\overrightarrow{H}}_{b}+\overrightarrow{H}(E),$$

where ${\overrightarrow{H}}_{d}$ is the magnetostatic field, ${\overrightarrow{H}}_{ex}$ is the exchange field, ${\overrightarrow{H}}_{a}$ is the anisotropy field, ${\overrightarrow{H}}_{b}$ is the external bias magnetic field. This formalism has been used to model the spin wave propagation in permalloy thin films with good agreement to experimental data^10^. We introduce a new term in Eq. (2) $\overrightarrow{H}(E)$ which accounts for the effect of electric field-controlled strain-mediated coupling. The coupling between the synthetic multiferroic and spin wave bus can be mathematically introduced using an additional magnetic field.

Without loss of generality, we restrict our consideration to a single type of spin wave: the magnetostatic surface spin waves also known as Damon-Eshbach spin waves. The dispersion of the magnetostatic surface spin wave can be expressed in an analytical form as follows^32^:

3 $$f=\gamma \sqrt{{H}_{eff}\cdot ({H}_{eff}+4\pi {M}_{s})+{(\frac{4\pi {M}_{s}}{2})}^{2}(1-{e}^{-2kd})},$$

where $\gamma$ = 2.8∙10^6^ Hz/Oe, *d* is the thickness of the film, and $4\pi {M}_{s}\,\,$is its saturation magnetization. There is a window in the *f*-*H* parameter space to propagate spin waves for any given *k*. Precession of magnetization obeys the Landau-Lifshitz-Gilbert equation which has a solution at frequencies and fields near resonance approximated by the Lorenz resonance curve^33^. The region of the *f*-*H* parameter allowing spin wave propagation is defined by the width of the spin wave resonance ΔH. Moving away from this region prevents spin wave propagation. Thus, by applying an electric field across the piezoelectric, we can switch between the spin wave conducting and non-conducting regimes. In Fig. 1(B), we show the output characteristic of the proposed modulator. The transmission is shown as a function of the applied electric field. It has a Lorentz line shape, where the maximum of the transmission occurs at the spin-wave resonance condition (e.g., the exact solution of Eq. (3)). The transmission decreases as the effective magnetic field is moved away from the resonance.

## Experimental data

**Figure 2:**
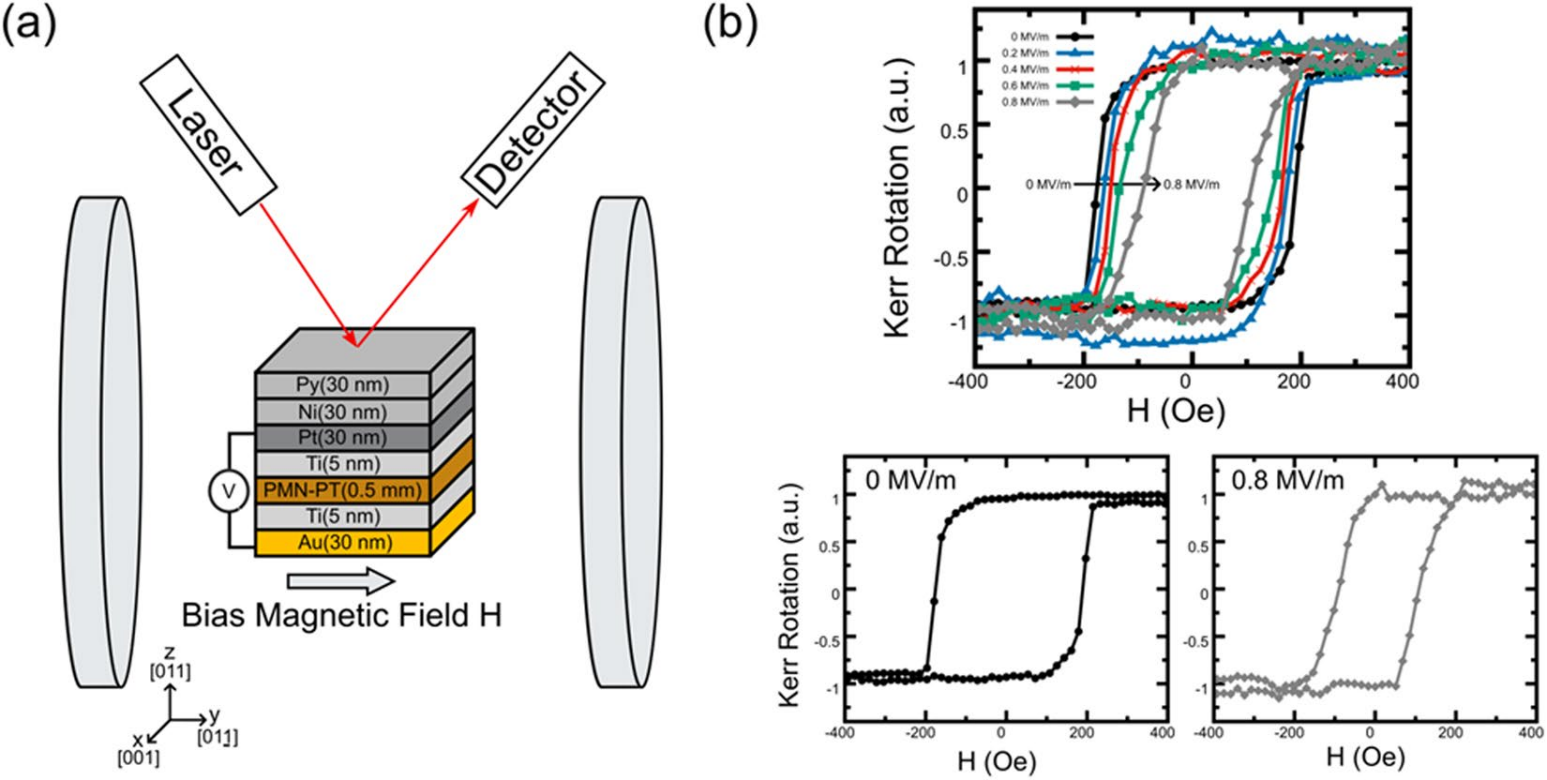
(**a**) Schematics of the PMN-PT/Ni samples and experimental setup for MOKE measurements. (**b**) Experimental data M-H loops obtained at different applied voltages.

Stress-mediated coupling between the piezoelectric and magnetostrictive materials is the base of the proposed modulator operation. The first set of experiments is aimed to show the effect of an electric field applied across the piezoelectric on the magnetic properties of the magnetostrictive material. Specifically, these effects are inferred from changes in the measured hysteresis loops of the uppermost magnetic layer. The cross section of the test structure is shown in Fig. 2(a). The sample was fabricated on a 10 mm × 10 mm × 0.5 mm single crystal (011) PMN-PT substrate sourced from TRS Technologies. From the bottom to the top, it consists of the following layers 30 nm Au, 5 nm Ti layer, 0.5 mm PMN-PT (011 single crystal cut), 5 nm Ti, 30 nm Pt, 30 nm Ni, and 30 nm Py. Prior to all metal depositions, the sample is cleaned with acetone/methanol/IPA followed by a 500 W O_2_ plasma clean. Ti is chosen as an adhesion layer for the Au and Pt electrodes. These electrodes are deposited so that the sample can be poled prior to deposition of the magnetic layers. In particular, a custom brass holder and voltage amplifier are used to apply an electric field to the sample for poling. Specifically, the applied electric field is linearly ramped from 0 MV/m to 0.8 MV/m over a 1 minute period and is then held constant for the same amount of time. Following this, the field is removed at the same rate it was applied. This is important because poling the sample after depositing the magnetic layers can lead to residual stresses that cannot be overcome with voltage-induced piezoelectric stresses. Pt is used because of its high electrical conductivity and superior resistance to oxidation. Hysteresis measurements of the sample are conducted using longitudinal magneto-optic Kerr effect (MOKE). It allows us to conclude on the sample magnetization by the changes in the reflected light polarization. The *M-H* loops are shown in Fig. 2, where the applied magnetic field is directed in-plane along the [011] crystallographic axis of the PMN-PT substrate. The applied magnetic field ranges between ± 400 Oe in 25 Oe increments. M-H curves are recorded for applied electric fields of 0 MV/m-0.8 MV/m in steps of 0.2 MV/m. For this sample, an 0.8 MV/m electric field produces a linear response with in-plain anisotropic strains of εx = 350 μm/m and εy = −1200 μm/m. The magnetization data is recorded using a standard lock-in technique. The hysteresis curves in Fig. 2 clearly show a coercive field dependence with applied electric field. In particular, at 0 MV/m the coercive field is 190 Oe and decreases to ~100 Oe with 0.8 MV/m. This modification to the materials anisotropy is caused by voltage-induced strain creating a magnetic easy axis in the Ni film orthogonal to the externally applied magnetic field.

**Figure 3:**
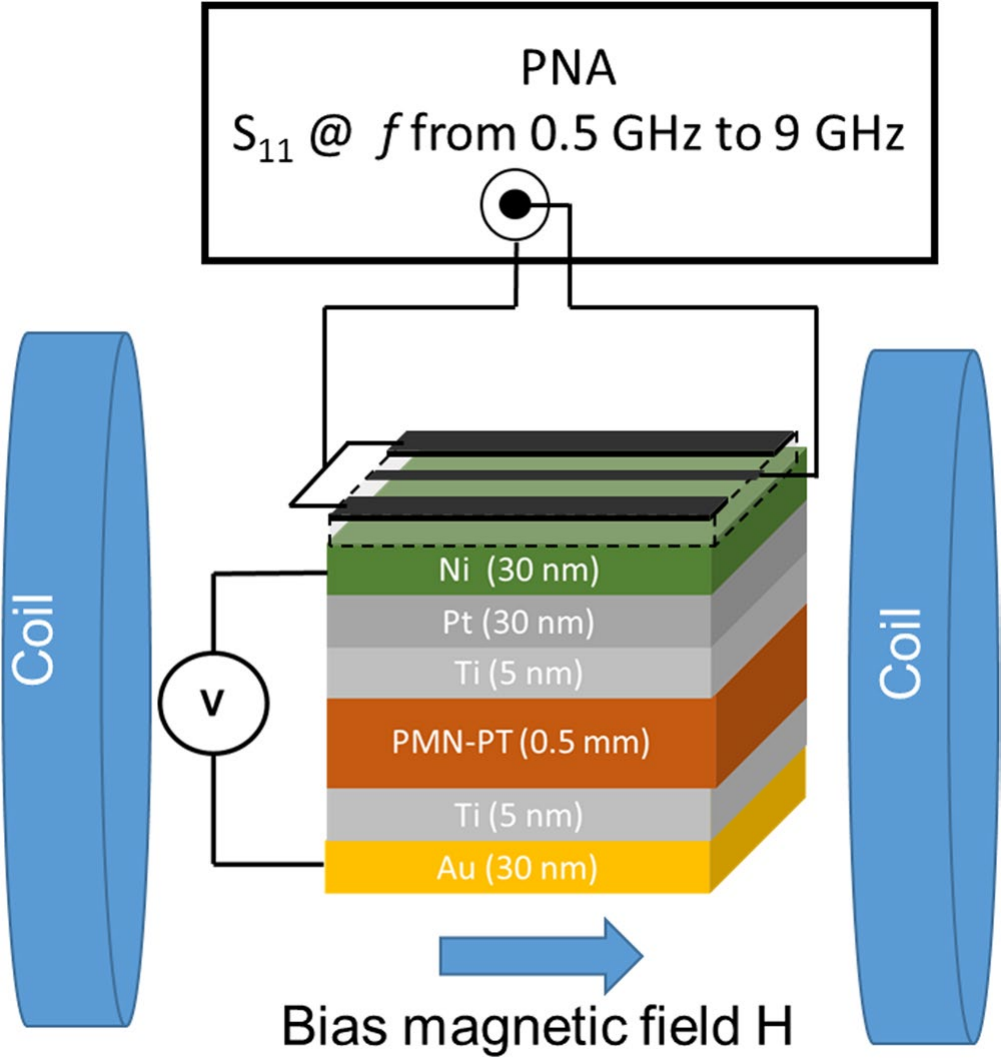
Schematics of the experimental setup for microwave absorption measurements. A microstrip antenna is mechanically attached to the sample. The antenna is connected to PNA to record microwave reflection S_11_ parameter from the sample.

**Figure 4:**
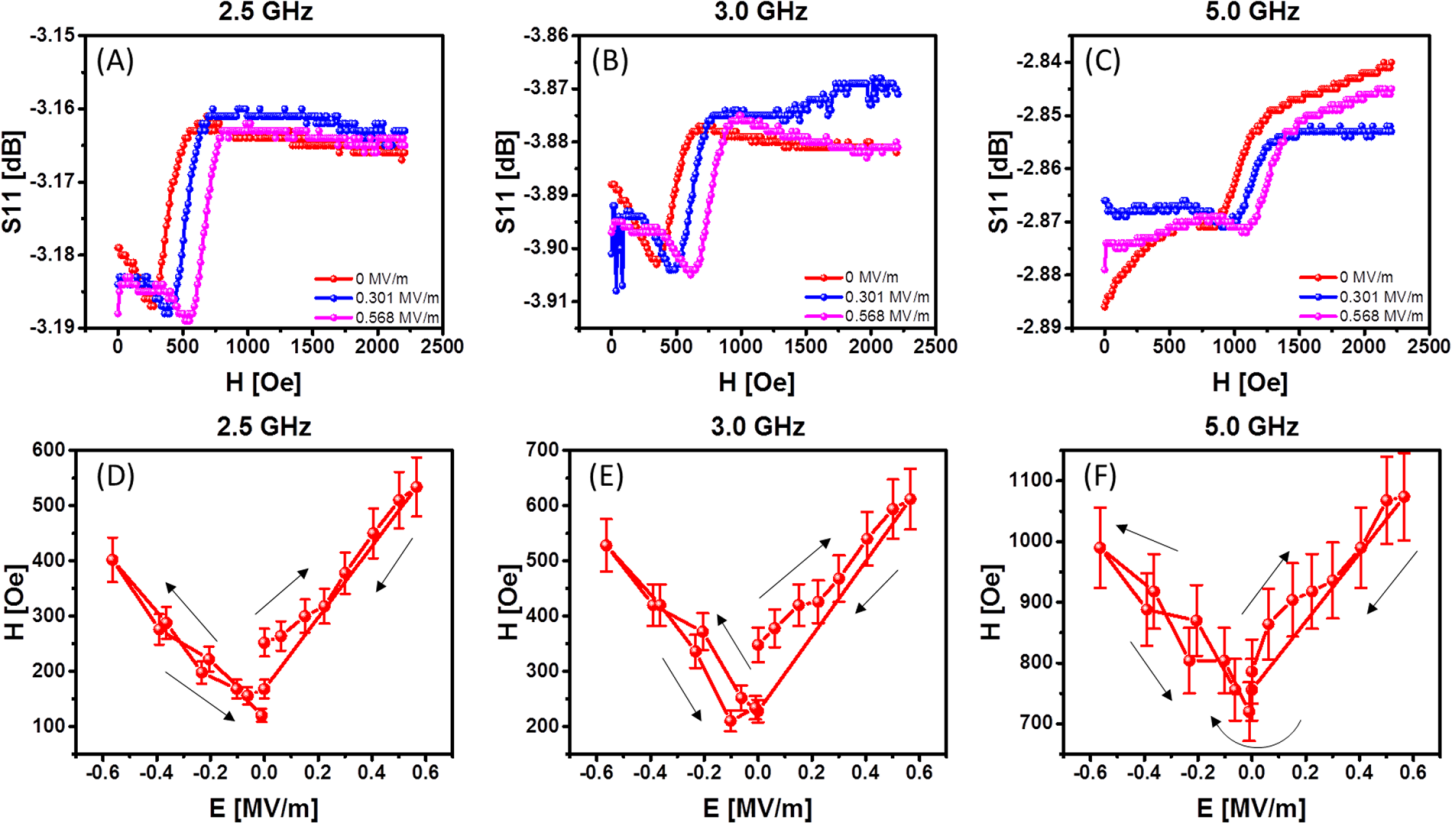
Experimental data on microwave reflection - S_11_ parameter. (**A**–**C**) S_11_ is shown as a function of the bias magnetic field for free selected frequencies 2.5 GHz, 3.0 GHz, and 5.0 GHz. The curves of different color correspond to the three electric fields: 0 MV/m, 0.301 MV/m, and 0.568 MV/m. (**D**–**F**) Traces of the microwave absorption position as a function of the applied electric field. The electric field follows the path: 0MV/m → +0.6MV/m → 0MV/m → −0.6MV/m → 0MV/m.

Next, we studied the magnetoelectric coupling between the piezoelectric and magnetostrictive layers by measuring microwave absorption in Ni as a function of the applied electric field to PMN-PT. The schematics of the experimental setup are shown in Fig. 3. The sample was placed inside an electromagnet GMW model 3472 − 70, pole cap 50 mm (2 inches) diameter tapered, which provides uniform bias magnetic field *ΔH/H* < 10^−4^ per 1 mm in the range from −2000 Oe to +2000 Oe. On the top of the sample, we mechanically placed a microstrip antenna. The width of the antenna is 100 μm, and the length is 5 mm. The antenna is connected to a programmable network analyzer (PNA) Keysight N5221A. Using this setup, we measured the S_11_ parameter, which indicates the microwave power reflected from the sample. Figure 4 presents a collection of experimental data on microwave reflection: S_11_ parameter. In Fig. 4(A–C), S_11_ parameter is shown as a function of the bias magnetic field for three selected frequencies: 2.5 GHz, 3.0 GHz, and 5.0 GHz. The dips in the S_11_ spectra correspond to the microwave absorption in the Ni layer (i.e., ferromagnetic resonance - FMR). There are three curves of different colors for each plot in Fig. 4(A–C). These curves correspond to the three different electric fields (0 MV/m, 0.301 MV/m, and 0.568 MV/m). There are two critical observations that need to be discussed based on the experimental data in Fig. 4(A–C). (i) The minima in S_11_ spectra shift to a higher magnetic field with increasing frequency. This trend is well explained by Eq. (3). (ii) The applying of an electric field increases the effective magnetic field ${H}_{eff}$ due to the effect of the stress-mediated coupling as expressed in Eq. (2). In Fig. 4(D–F), we show experimental data tracing the position of the peak of microwave absorption as a function of the applied electric field. The starting point corresponds to 0 MV/m electric field. Then, the electric field follows the path: 0 MV/m → +0.6 MV/m → 0 MV/m → −0.6 MV/m → 0 MV/m. As one can observe from Fig. 4(D–F), the absorption peak in the S_11_ spectra follows a closed loop as a function of the applied electric field. The absorption peak shift exceeds 250 Oe in response to ±0.6 MV/m electric field. The error bars in Fig. 4(D–F) reflect the uncertainty in the S_11_ minima detection, which can be concluded from Fig. 4(A–C). There is an asymmetry in the position of the peak of microwave absorption showing a higher response at +E compared to −E. We attribute it to the sample tilting in magnetic field. In general, both the piezoelectric and magnetic properties of the sample exhibit a reversible change, which should be taken into consideration.

**Figure 5:**
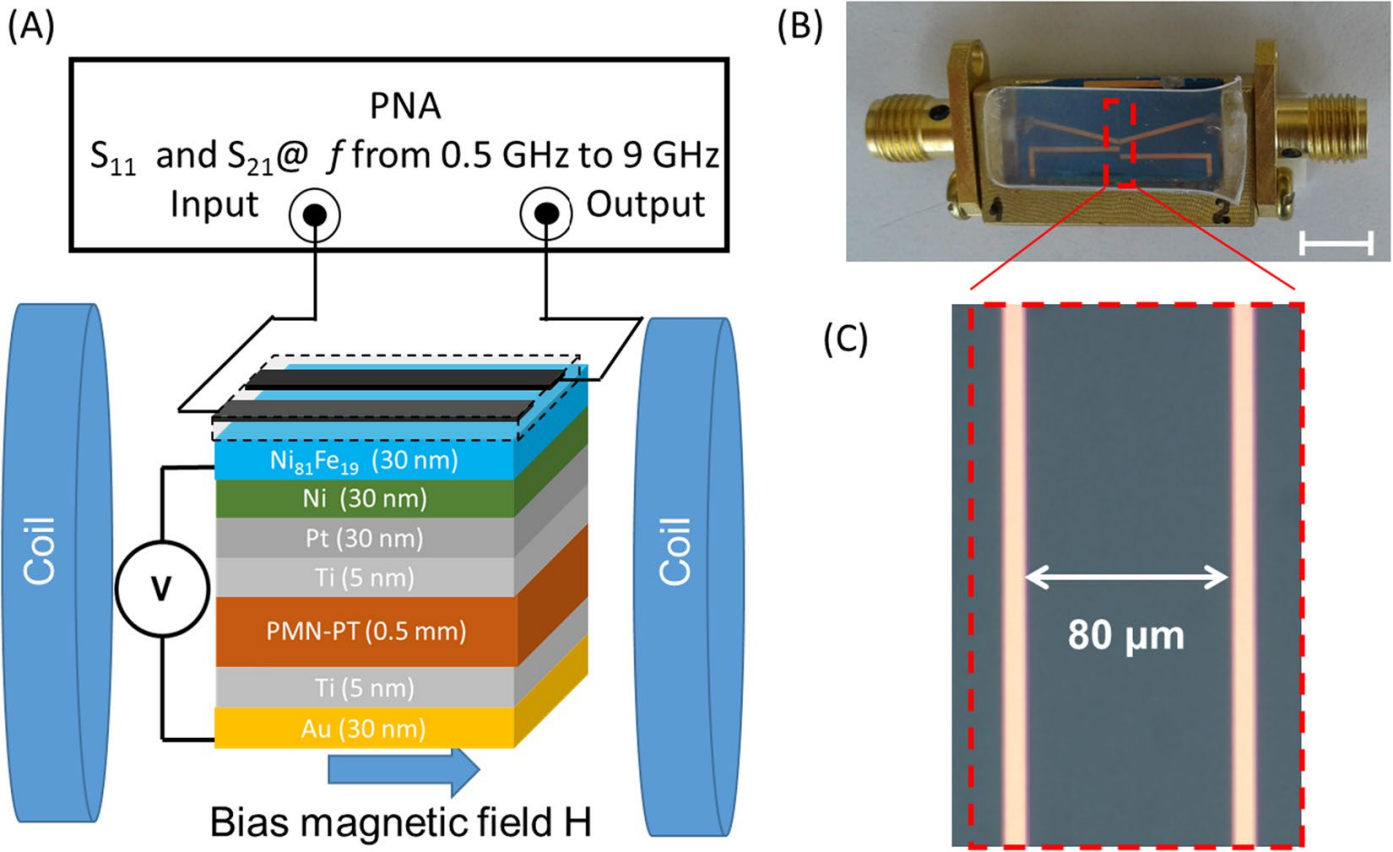
(**A**) Schematics of the experimental setup for spin wave excitation and detection in PMN-PT/Ni/Py sample. There is a set of two micro-antennas mechanically attached to the sample. One antenna is to excite spin waves, the second antenna is to detect the inductive voltage produced by the propagating spin waves. (**B**) Photo of the device packaged. The antennas are connected to PNA to measure S_21_ parameter. (**C**) Enlarged photo of the active area with antennas. The distance between the antennas is 80 μm.

Then, we studied spin wave excitation and propagation in a structure with Ni_81_Fe_19_ (Py) spin wave bus. There are several reasons for choosing the combination of Py spin wave bus with Ni magnetostrictive layer. In contrast to Ni, Py possesses much lower spin wave damping which produces spin waves propagating over tens of microns at room temperature. Also, from the fabrication point of view, Py is well matched to Ni due to the large nickel content. The layer of magnetostrictive nickel is needed for voltage-controlled modulation as Py films maintain near-zero magnetostriction with film thicknesses above 7 nm^34^. The structure of the sample is shown in Fig. 5. The core of the structure is the same as in Fig. 3 except a 30 nm Py layer fabricated on top of Ni layer

**Figure 6:**
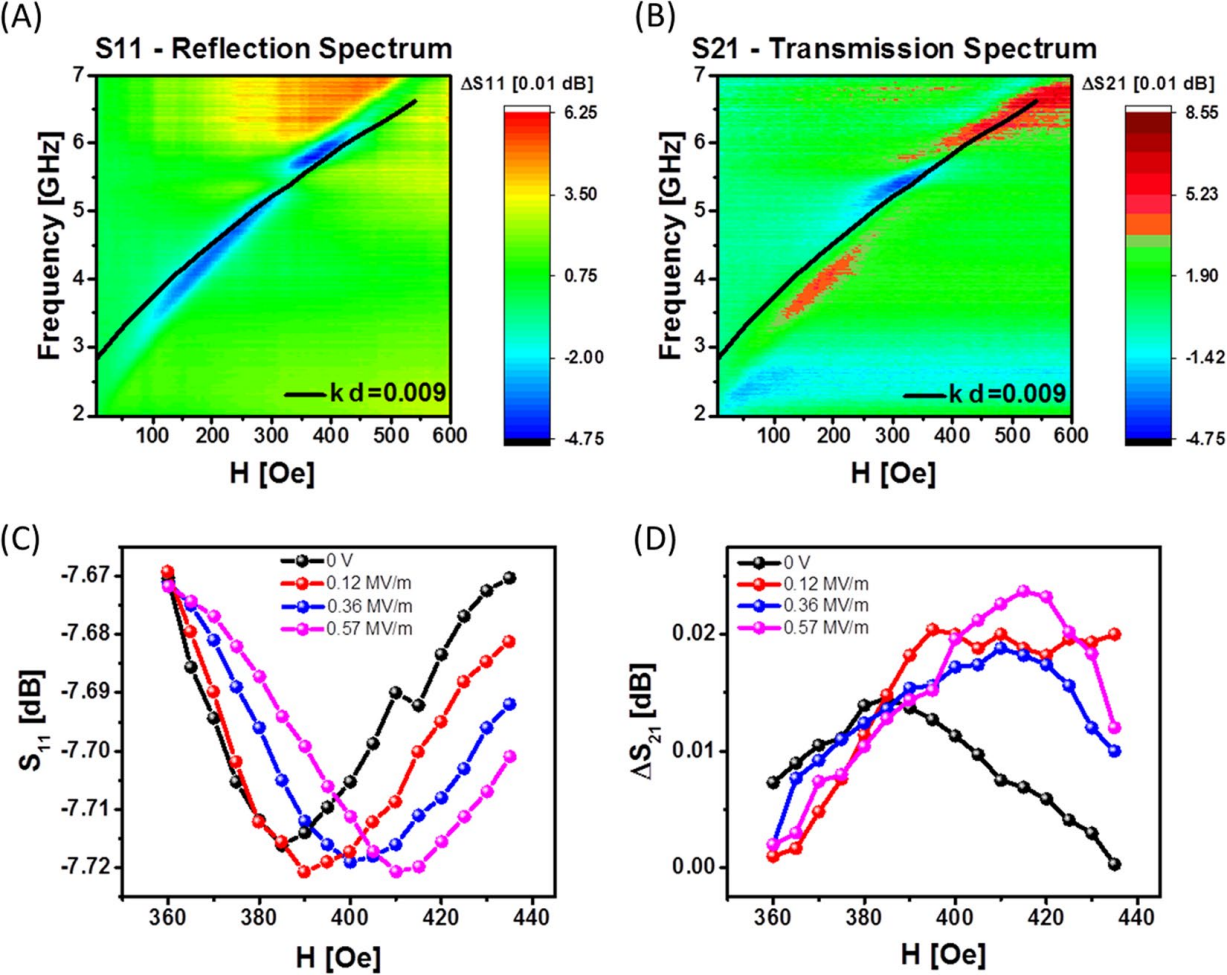
(**A**) Color map showing the S_11_ parameter in the frequency range *f* from 2.0 GHz to 7.0 GHz and the bias magnetic field *H* from 0 Oe to 600 Oe. The magnetic field is directed in-plane and orthogonal to the spin wave propagation. (**B**) Color map showing the S_21_ parameter in the same frequency and bias magnetic field ranges. The black curve in (**A**,**B**) shows the results of fitting by Eq. (3). (**C**) Microwave reflection S_11_ as a function of the bias magnetic field *H* at *f* = 6.0 GHz. The four curves of different color show S_11_ variation at the applied electric field *E*: 0 MV/m, 0.12 MV/m, 0.36 MV/m, and 0.57 MV/m. The minima in the S_11_ spectra correspond to the microwave power absorption. (**D**) Transmission parameter S_21_ as a function of the bias magnetic field *H* at *f* = 6.0 GHz. The four curves of different color depict S_21_ parameter at the applied electric field *E*: 0 MV/m, 0.12 MV/m, 0.36 MV/m, and 0.57 MV/m. The maxima in the S_21_ spectra correspond to the spin wave propagation.

In order to study spin wave transport, we used a modified multi-antenna structure as shown in Fig. 5. One of the conducting contours is used for spin wave excitation (e.g., the one connected to the PNA input) and the second contour (e.g., connected to the PNA output) is used for detecting the inductive voltage, which is produced by the propagating spin-waves. The wave vector *k* of the excited spin waves is mainly defined by the width of the antenna (i.e., *k∙w ≈ π*)^35,36^. For a 10 μm wide excitation wire, the estimated *k* is about π/10 µm ≈ 0.3 µm^−1^ or 0.3∙10^4^ cm^−1^ (*kd* = 0.009 « 1). The distance between the centers of the excitation and detection antennas is 80 μm. A more detailed description of the inductive measurement technique can be found in ref.^10^. In Fig. 6, we present the collection of experimental data showing S_11_ and S_21_ parameters measured at different frequencies and bias magnetic fields. The color map in Fig. 6(A) shows S_11_ parameter in the frequency range from 2.0 GHz to 7.0 GHz and the bias magnetic field from 0 Oe to 600 Oe. The bias magnetic field is directed in-plane and orthogonal to the spin wave propagation. The red color in Fig. 6(A) corresponds to the maximum reflection while the blue color depicts the region in the *f*-*H* space corresponding to the microwave absorption. The color plot in Fig. 6(B) shows S_21_ parameter collected in the same frequency/magnetic field range. The red color in Fig. 6(B) corresponds to the maximum signal transmission (e.g., via the propagating spin waves). In order to exclude the effect of the direct coupling between the antennas, the transmission is subtracted to the value at zero bias magnetic field: S_21_ = S_21_(H) − S_21_(H = 0). The black curve in Fig. 6(A,B) shows the results of fitting by Eq. (3) using the following parameters: 4*πM*_*s*_ = 7800 G, *kd* = 0.009. The results of fitting match well experimental data for both reflection and transmission as one can see on Fig. 6(A,B).

In Fig. 6(C), we present experimental data showing the change of the microwave reflection due to the applied electric field. The data are collected at *f* = 6.0 GHz as the maximum of the signal absorption/transmission was detected near this particular frequency. There are four curves of different color depicting S_11_ parameter as a function the applied electric field (e.g., 0 MV/m, 0.12 MV/m, 0.36 MV/m, and 0.57 MV/m). The dip in the S_11_ spectra corresponds to the microwave power absorption. Similar to the previously reported data in Fig. 4, the applied electric field shifts the absorption peak towards a higher magnetic field. At the same time, the shift is smaller compared to the structure without Py layer. For instance, the applying of 0.57 MV/m shifts the absorption peak in PMN-PT/Ni by 250 Oe, while the same applied electric field results in only a 20 Oe shift in the PMN-PT/Ni/Py sample. We attribute this difference to the effect of the interlayer magnetostatic coupling^37,38^. The dynamical pinning between thin layers of nickel on epitaxial permalloy films was investigated by stationary spin wave resonances^39^. The pinning originates from the exchange and magnetostatic interactions affecting magnetizations of the ferromagnetic films. In Fig. 6(D), we present experimental data showing the change of the transmission between the antennas due to the applied electric field. The input microwave power is 10 dBm in all experiments. There are four curves of different color depicting S_21_ parameter at different applied electric field (e.g., 0 MV/m, 0.12 MV/m, 0.36 MV/m, and 0.57 MV/m). The maxima in the S_21_ spectra correspond to the spin wave propagation. The obtained shift towards higher frequency is well consistent with the similar trend in S_11_ parameter. The applying of an electric field *E* across the piezoelectric layer is equivalent to the change of the effective magnetic field ${H}_{eff}$.

**Figure 7:**
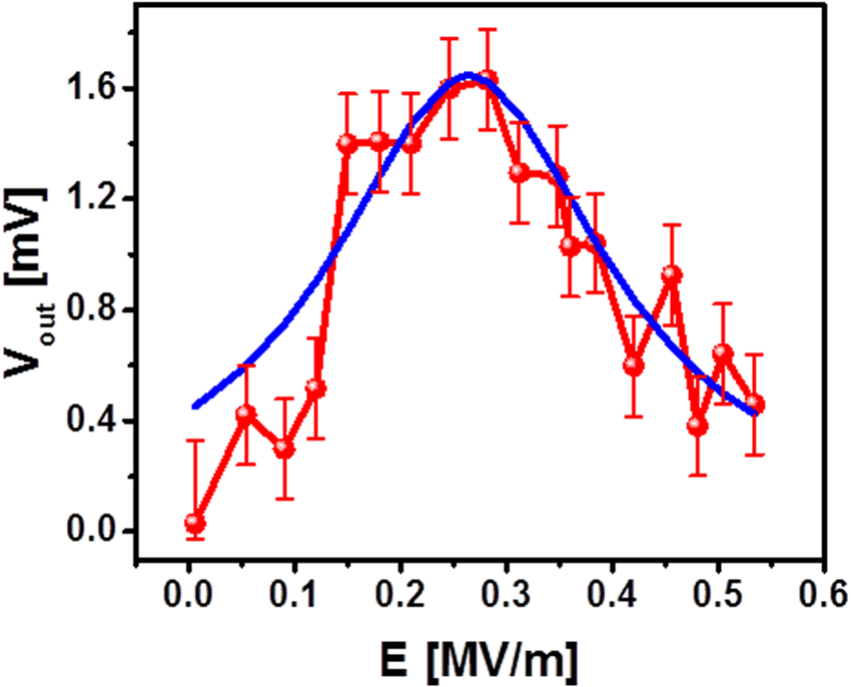
Experimental data showing the output characteristics of the spin wave modulator. Red markers and red curve show the inductive voltage in mV produced by the propagating spin-waves. The data are taken at the bias magnetic field of 390 Oe and the operational frequency *f* = 6.0 GHz. The blue curve shows the results of fitting by Lorentz lineshape. The distance between the excitation and detection antennas is 80 μm. All measurements are done at room temperature.

Finally, we extracted experimental data demonstrating the change of the inductive voltage V_out_ as a function of the applied electric field *E* as shown in Fig. 7. The red markers depict the inductive voltage in mV produced by the propagating spin-waves. The data are collected at room temperature, with a constant bias magnetic field of 390 Oe directed orthogonal to the spin wave propagation, and the operational frequency of 6.0 GHz. We intentionally chose the bias magnetic field to be about 20 Oe away from the spin wave resonance. The experiment starts with zero applied electric field *E* = 0, which corresponds to the non-conducting spin wave regime. The output inductive voltage is close to zero within the thermal noise floor. An increase of the electric field strength is mathematically equivalent to the applying of an external magnetic field, which makes spin wave propagation possible. The peak of the spin-wave transmission corresponding to 1.5 mV output inductive voltage takes place around 0.3 MV/m of the applied electric field. The further increase of the electric field leads to the decrease of the output voltage. The decrease of the output inductive voltage reflects the decrease of the spin wave amplitude as the system moves away from the spin wave resonance. The maximum applied electric field is 0.6 MV/m, which is limited by the experimental capabilities. We want to stress that the data in Fig. 7 are only related to the inductive voltage produced by the spin waves. In order to exclude the direct coupling and other effects not-related to spin wave transport, we made a subtraction to zero bias magnetic field Δ*S*_21_ = *S*_21_(*H*) − *S*_21_(*H* = 0). Only a small percent of the total output power is related to the spin wave signal (*e*.*g*., Δ*S*_21_ ~ 0.01 *db*) which also limit the accuracy of the spin wave measurements. The error bars in Fig. 7 reflect possible variation of the output signal depending on the absolute value of *S*_21_(*H*). The data shows a prominent modulation of the inductive voltage produced by the propagating spin-waves as a function of the applied electric field. The transmission decreases by more than 3 times compared to the maximum value by changing the electric field ±0.3 MV/m. The blue curve in Fig. 7 shows the results of fitting using Lorentz line shape (i.e., V_out_(*E*) using V_out_(max) = 1.64 mV, and *E*(max) = 0.264 MV/m. The results of fitting are in good qualitative agreement with our expected output characteristic shown in Fig. 1(B).

## Discussion

There are several unique properties of the described modulator to be outlined and discussed. First of all, spin wave propagation is controlled by applying an electric field. The latter produces minimal power consumption compared to conventional methods. The artificial multiferroic element is analogous to a parallel plate capacitor, where one of the plates is made of a magnetostrictive material. The charging/discharging of the capacitor affects the magnetic properties of the magnetostrictive material via the stress-mediated coupling. A relatively weak electric field ${E}_{sw}$ (e.g., 0.6 MV/m for PMN-PT/Ni/Py structure) is required to switch between the spin-wave conducting and spin wave non-conducting regimes. The energy dissipated in the multiferroic element ${\rm{{\rm E}}}$ can be estimated as follows:

4 $${\rm{{\rm E}}}=\frac{1}{Q}\frac{C{V}^{2}}{2}=\frac{{\varepsilon }_{0}\varepsilon S{d}_{e}}{2Q}{E}_{sw}^{2},$$

where *C* is the capacity of the multiferroic element, *V* is the voltage applied, *Q* is the quality factor of the piezoelectric oscillator, ${\varepsilon }_{0}=8.854\times {10}^{-12}\,{\rm{F}}\cdot {{\rm{m}}}^{-1}$ is the electric constant, *ε* is the dielectric permittivity of the piezoelectric, *S* is the plate area, and *d*_*e*_ is the thickness of the piezoelectric layer. Assuming the switching electric field *E*_*sw*_ constant, one can estimate energy dissipation in sub-micrometer size devices. Taking *ε* = 2000, *S* = 100 nm × 100 nm*; d*_*e*_ = 20 nm, and *Q* = *1*, the *energy dissipated per one modulation is less than 10 aJ neglecting line losses and substrate clamping*. More details on the power dissipation in the synthetic multiferroic element can be found in refs^28,40^. Ultra-low power consumption represents a significant advantage of using magneto-electric coupling in magnetic logic devices when compared to conventional approaches^4^. The proposed modulator may be more efficient than the VCMA-based devices^19,20^ as it requires much weaker electric field for operation.

The modulation frequency of the multiferroic–based modulator is limited by the speed of the stress-mediated coupling. It should be noted that the most experiments on bulk magnetoelectric coupling (e.g., ref.^25^) are taken at relatively low frequencies below 1 kHz. However, high frequency (1 GHz) PZT nanopowder based oscillators have been demonstrated^41^. Furthermore, experimental data reported in ref.^29^ show up to 6 GHz spin wave generation by the PMN-PT/Ni/Py synthetic multiferroic structure. These experimental results provide some insight on the possibility of high-frequency spin wave modulation. To the best of our knowledge, there is no theoretical model describing the high-frequency dynamics of composite multiferroics. The development of RF-frequency operating magneto-electric cells is important to a variety of magnetic devices including spin wave amplifiers^28^.

Modulation depth *η* is a parameter which shows how much the modulated variable of the carrier signal varies around its unmodulated level. In our case, we consider the spin wave as a carrier signal and define spin wave amplitude as a signal variable. The amplitude of the inductive voltage *V* is directly proportional to the amplitude of the spin wave^42^. Thus, we define the modulation depth *η* as follows:

5 $$\eta =\frac{{A}_{max}}{A(E)}=\frac{{V}_{max}}{V(E)},$$

where ${A}_{max}$ is the maximum amplitude of the unmodulated spin wave at resonance conditions, $A(E)$ is the spin wave amplitude at the electric field corresponding to the non-conducting regime, ${V}_{max}$ is the maximum inductive voltage produced by the unmodulated spin wave, $V(E)$ is the inductive voltage produced by the modulated spin wave. In theory, the modulation depth goes to infinity as the amplitude of the modulated signal approaches zero. The maximum practically feasible modulation depth is limited by the thermal noise $\eta ={V}_{max}/{V}_{noise}$. Taking the results of Lorentzian fitting presented in Fig. 6(B) ${V}_{max}=1.5\,mV$ and $V(\pm 0.3MV/m)=0.5\,mV$, we estimate $\,\eta =300 \%$. The modulation takes place 80 μm away from the excitation port *at room temperature*. For comparison, the oscillatory conductance modulation in InAs-based spin-FET *η* < 1% was observed at T = 1.8 K by non-local measurements with the channel length of 1.65 μm^8^. The demonstration of the spin wave modulator shows a possible route for spin-based devices development free of the constrains associated with short spin diffusion length. Based on our S_21_ amplitude and phase measurements, we estimated the spin wave group velocity, which is about 5∙10^5^ cm/s. The decay length is about 8.4 μm. These estimates are consistent with the available data obtained for sub-micrometer thick Py films (e.g. velocity 8.6∙10^5^ cm/s and the decay length of 7.1 μm in 30 nm reported in ref.^43^, and the decay length of 15.44 μm reported in ref.^44^).

The further development of the spin wave modulator is mainly associated with the scaling down of the multiferroic element. One of the major questions is related to the effect of scaling to the switching electric field ${E}_{sw}$ required for spin wave modulation. The lack of experimental data does not allow us to conclude whether the switching electric field will be the same in sub-micrometer thick piezoelectric/magnetostrictive structures. Scaling down the dimensions of the multiferroic element with constant ${E}_{sw}$ would benefit its operation in all figures of merit. However, if the piezoelectric layer is reduced to a thin film deposited on a Si substrate, surface clamping effects become important and may reduce the effective strain generated in the ferromagnetic material. Recent studies have demonstrated an approach to overcome this substrate clamping issue using patterned surface electrodes. The patterned surface electrodes produce localized strain that is sufficient for controlling the magnetic anisotropy of nanoelements^45^. The structure of the synthetic multiferroic element can be further optimized by engineering the thickness ratio and the geometry of the piezoelectric-magnetostrictive layers. In general, the magnetostrictive material itself can be utilized as a spin wave bus. However, most conventional magnetostrictive materials (e.g., Ni, Ni_50_Fe_50_) have relatively high spin wave damping. It will be important to study the effect of the strain-mediated coupling on the different types of spin waves (e.g., backward volume magnetostatic spin waves). Overall, the described modulator may be of great value for the spin-wave based logic devices due to its ultra-low power consumption. Also, synthetic multiferroic elements may provide a link among the conventional electronic and magnonic devices.

## Conclusion

We described a spin wave modulator, which operation is based on the stress-mediated coupling between piezoelectric and magnetostrictive materials. Spin wave propagation in ferromagnetic spin wave bus is controlled by applying an electric field across the piezoelectric layer. By applying an electric field to the piezoelectric layer, the stress is produced. In turn, the stress changes the direction of the easy axis in the magnetostrictive layer and affects spin wave transport. We presented experimental data on a PMN-PT/Ni/Py structure. The switching between the spin wave conducting and non-conducting states is achieved by applying of ±0.3 MV/m electric field. We report over 300% modulation depth at the distance of 80 μm away from the excitation port at room temperature. The demonstration of the spin wave modulator shows a possible solution to the problems associated with a short spin diffusion length. The proposed magnetoelectric modulator can be utilized in spin-wave logic and memory devices by offering ultra-low power consumption and fast modulation speed.
